# Differences in Ureolytic Bacterial Composition between the Rumen Digesta and Rumen Wall Based on *ureC* Gene Classification

**DOI:** 10.3389/fmicb.2017.00385

**Published:** 2017-03-07

**Authors:** Di Jin, Shengguo Zhao, Nan Zheng, Dengpan Bu, Yves Beckers, Stuart E. Denman, Christopher S. McSweeney, Jiaqi Wang

**Affiliations:** ^1^State Key Laboratory of Animal Nutrition, Institute of Animal Science, Chinese Academy of Agricultural SciencesBeijing, China; ^2^Precision Livestock and Nutrition Unit, Gembloux Agro-Bio Tech, University of LiègeGembloux, Belgium; ^3^Laboratory of Quality and Safety Risk Assessment for Dairy Products of Ministry of Agriculture (Beijing), Institute of Animal Science, Chinese Academy of Agricultural SciencesBeijing, China; ^4^Commonwealth Scientific and Industrial Research Organisation, Queensland Bioscience Precinct, St. LuciaQLD, Australia

**Keywords:** rumen digesta, rumen wall, ureolytic bacteria, *ureC* gene, difference

## Abstract

Ureolytic bacteria are key organisms in the rumen producing urease enzymes to catalyze the breakdown of urea to ammonia for the synthesis of microbial protein. However, little is known about the diversity and distribution of rumen ureolytic microorganisms. The urease gene (*ureC*) has been the target gene of choice for analysis of the urea-degrading microorganisms in various environments. In this study, we investigated the predominant *ureC* genes of the ureolytic bacteria in the rumen of dairy cows using high-throughput sequencing. Six dairy cows with rumen fistulas were assigned to a two-period cross-over trial. A control group (*n* = 3) were fed a total mixed ration without urea and the treatment group (*n* = 3) were fed rations plus 180 g urea per cow per day at three separate times. Rumen bacterial samples from liquid and solid digesta and rumen wall fractions were collected for *ureC* gene amplification and sequencing using Miseq. The wall-adherent bacteria (WAB) had a distinct ureolytic bacterial profile compared to the solid-adherent bacteria (SAB) and liquid-associated bacteria (LAB) but more than 55% of the *ureC* sequences did not affiliate with any known taxonomically assigned urease genes. Diversity analysis of the *ureC* genes showed that the Shannon and Chao1 indices for the rumen WAB was lower than those observed for the SAB and LAB (*P* < 0.01). The most abundant *ureC* genes were affiliated with Methylococcaceae, Clostridiaceae, Paenibacillaceae, Helicobacteraceae, and Methylophilaceae families. Compared with the rumen LAB and SAB, relative abundance of the OTUs affiliated with *Methylophilus* and *Marinobacter* genera were significantly higher (*P* < 0.05) in the WAB. Supplementation with urea did not alter the composition of the detected ureolytic bacteria. This study has identified significant populations of ureolytic WAB representing genera that have not been recognized or studied previously in the rumen. The taxonomic classification of rumen *ureC* genes in the dairy cow indicates that the majority of ureolytic bacteria are yet to be identified. This survey has expanded our knowledge of *ureC* gene information relating to the rumen ureolytic microbial community, and provides a basis for obtaining regulatory targets of ureolytic bacteria to moderate urea hydrolysis in the rumen.

## Introduction

Urea is used commonly as a non-protein nitrogen source in the diet of ruminants as an economical replacement for feed proteins (Kertz, 2010). Rumen ureolytic bacteria produce ureases which catalyze the breakdown of urea to ammonia and carbon dioxide (Owens et al., 1980). The ammonia from urea can be assimilated by many rumen bacteria for synthesis of microbial protein required for animal growth and thus partially replaces feed protein as a N source in the diet of the ruminant (Milton et al., 1997). Nowadays, urea, as a highly rumen-degradable nitrogen source, has been included in the rations of ruminants to supply adequate amounts of nitrogen for microbial protein synthesis and improve ruminal fermentation (Wagner et al., 2010; Ceconi et al., 2015). However, urea hydrolysis to ammonia often exceeds the rate of ammonia utilization, which leads to poor efficiency of urea utilization in the rumen (Patra, 2015).

Following extensive research on the utilization of urea as a replacement for protein in ruminant diets, interest has focused on urea-hydrolyzing microbes for a better understanding of urea metabolism in the rumen (Cook, 1976; Wozny et al., 1977; On et al., 1998). Kakimoto et al. (1989) assayed about 16,000 isolates from animal feces and intestines for the production of acid urease and found that most of the selected strains belonged to the genera *Streptococcus* and *Lactobacillus*. In a similar study by Lauková and Koniarová (1994), they tested 909 strains from the rumen of 104 domestic and wild ruminants for urease activity, and their results showed that some *Selenomonas ruminantium* strains and *lactobacilli* demonstrated medium urease activity and most of the *Enterococcus faecium* and all of the *E. faecalis* isolates expressed urease activity. In addition, *Howardella ureilytica*, a Gram-positive bacterium has been isolated from the rumen fluid of a sheep, which was strongly ureolytic and generated ATP through the hydrolysis of urea (Cook et al., 2007). All these above studies were conducted using culture based methods. However, most rumen microorganisms remain uncultured (Edwards et al., 2004), and therefore little is known about the identities and diversity of rumen organisms capable of urea hydrolysis.

Ureases synthesized by ureolytic bacteria are commonly composed of two or three subunits (*ureA, ureB*, and *ureC*) and require up to several accessory proteins (such as *ureD, ureE, ureF, ureG, ureH*, and *ureI*) for activation (Mobley et al., 1995). The *ureC* subunit is the largest of the genes encoding urease functional subunits and contains several highly conserved regions that are suitable as PCR priming sites. Primers for *ureC* gene have been designed and applied for analysis of the urea-degrading microorganisms in various environments, including the open ocean (Collier et al., 2009), sponges (Su et al., 2013), and soil (Singh et al., 2009). We have previously studied the rumen ureolytic bacteria using an *ureC* gene clone library, and found that ureolytic bacterial composition in the rumen is distinct from that in other environments (Zhao et al., 2015). So it is of great interest to investigate the unknown rumen ureolytic bacteria in further detail. In this study, we investigated the diversity of the *ureC* genes in different rumen fractions, and revealed the predominant *ureC* gene operational taxonomic units (OTUs) in the rumen of dairy cows using Miseq sequencing. Animals were also fed with urea to determine if supplementation alters the growth of some populations of ureolytic bacteria or alters the ureolytic community composition.

## Materials and Methods

### Animals and Diets

Six Chinese Holstein dairy cows (550 ± 50 kg bodyweight and 100 ± 21 days in milk) fitted with ruminal cannulas were used in a two-period cross-over trial. All cows were fed *ad libitum* the same total mixed ration (TMR) for 2 weeks prior to the study. Cows were divided into the following groups: Urea group received 180 g daily urea as a stimulator for ureolytic bacteria, and the control group, which did not receive urea supplementation. The experiment proceeded for a period of 21 days, followed by a 14 days washout period, after which the intervention was crossed. This cross-over was used to assess the functional diversity of the bacterial communities. Each day, the total urea was separated into three parts (70, 55, and 55 g for morning, afternoon, and evening feeding, respectively) and was packaged in filter paper to prevent ammonia toxicity from rapid hydrolysis. Urea was added into the rumen through a cannula during each feeding. All cows were kept in individual pens with free access to water and were fed TMR three times daily (7:00, 14:00, and 19:00). The TMR consists primarily of alfalfa hay (28.4%), corn silage (26.7%), corn (22.6%), and soybean meal (11.8%) [Dry matter (DM) basis] (Supplementary Table S1). Animals involved in this study were cared for according to the principles of the Chinese Academy of Agricultural Sciences Animal Care and Use Committee (Beijing, China).

### Rumen Sampling and Sample Detection

For each animal, samples of rumen contents (solid and liquid phase) and rumen papilla were obtained on days 20 and 21 of the experiment shortly before morning feeding (0 h) and at 2, 4, and 6 h after morning feeding. Essentially, approximately 300 g of mixed rumen contents were taken from each cow through the rumen fistula. Rumen samples were filtered with four layers of cheesecloth, allowing the separation of rumen solids from the liquid fraction. The aliquots of the liquid fraction were dispensed into centrifuge tubes. Approximately 100 μL of hydrochloric acid (6 mol L^-1^) was added to 10 mL of filtered rumen fluid for detection of urea nitrogen (Urea-N) and ammonia nitrogen (NH_3_-N). The solid fraction was washed with 50 mL of ice-cold phosphate-buffered saline (PBS) twice and residues were kept. Rumen papillae samples were collected by scraping with a spatula from different rumen locations (the front-, middle- and post-ventral sac) via the rumen cannula and washed twice in ice-cold PBS (Petri et al., 2013). All rumen samples were snap frozen in liquid nitrogen and stored at -80°C for further analysis.

Rumen fluid samples were centrifuged (13, 000 × *g* at 4°C for 15 min) and supernatants were stored at -20°C until analyzed. NH_3_-N concentration was determined by using an adaptation of the method based on the Berthelot (phenol-hypochlorite) reaction (Broderick and Kang, 1980). Urea-N concentration was determined by the diacetyl monoxime method using a commercial kit (Nanjing Jiancheng Co., Nanjing, China). Urease activity was evaluated on total rumen microbial protein extracts by measuring the amount of ammonia released from urea according to Zhao et al. (2015). One unit of urease activity was defined as 1 μmol of ammonia released per min per mg microbial cytoplasmic protein.

### Microbial DNA Extraction

The rumen contents and papilla samples collected at 2 h after morning feeding were chosen for DNA extraction based on the high urea hydrolysis rates at this time. Rumen liquid fraction samples (1 ml) were centrifuged at 350 × *g* for at 4°C 10 min to remove the feed residue, and the supernatant were centrifuged at 16 000 × *g* at 4°C for 15 min to collect the liquid-associated bacteria (LAB). Approximate 0.5 g thawed rumen papilla and 0.5 g solid fraction was directly used for solid-associated bacteria (SAB) and wall-associated bacteria (WAB) DNA extraction, respectively. Total DNA of bacteria was extracted using cetyltrimethylammonium bromide (CTAB) plus bead beating method (Minas et al., 2011). Briefly, samples from each fraction was homogenized with 0.5 g zirconium beads (0.5 mm diameter) and 800 μL CTAB buffer (100 mM Tris-HCl, pH 8.0; 1.4 M NaCl; 20 mM EDTA; 2% CTAB) using a Mixer Mill MM 400 (Retsch, Haan, Germany) with vibrational frequency of 1800 min^-1^ and grinding time of 60 s. Then samples were incubated at 70°C for 20 min and centrifuged at 13, 000 × *g* for 10 min, and the supernatant was mixed with 600 μL phenol-chloroform-isoamyl alcohol (volume 25:24:1). The upper layer was transferred to new tube and mixed with 0.8 times volume of isopropanol to precipitate DNA. Extracted DNA was qualitatively assessed by agarose gel electrophoresis and quantified using a Nanodrop^TM^ spectrometer (Thermo Scientific, USA). DNA was diluted to a concentration of 50 ng μL^-1^, and was used as templates for amplification in the following PCRs.

### PCR Amplification of Urease Genes (*ureC*) and Illumina Sequencing

Urease (*ureC*) genes were amplified with the modified primer set, UreC-F 5′-barcode-TGGGCCTTAAAATHCAYGARGAYTGGG-3′ and UreC-R 5′-GGTGGTGGCACACCATNANCATRTC-3′ (Reed, 2001), where the barcode is an eight-base sequence unique to each sample. Reactions were performed in a MyCycler Thermal Cycler (Bio-Rad, USA) using a 50 μL mixture containing 5 μL 10 × PCR buffer (Invitrogen, Carlsbad, CA, USA), 1.5 μL MgCl_2_ (50 mM), 1 μL dNTP mixture (10 mM), 1.5 μL each forward and reverse primer (10 μM), 0.4 μL Platinum Taq DNA polymerase (Invitrogen), 2 μL rumen microbial DNA (100 ng μL^-1^), and 37.1 μL sterile ddH_2_O. PCR amplification began with a 5 min denaturing step at 94°C, followed by 30 cycles at 94°C for 30 s, 50°C for 30 s, and 72 °C for 30 s; extension was achieved at 72°C for 15 min. PCR amplicons of approximately 324 bp were extracted from 2% agarose gels and purified using the AxyPrep DNA Gel Extraction Kit (Axygen Biosciences, Union, CA, USA) according to the manufacturer’s instructions and quantified using QuantiFluor^TM^-ST (Promega US, Madison, WI, USA). Purified amplicons were pooled in equimolar and paired-end sequenced (2 × 300) on an Illumina MiSeq platform according to the standard protocols.

### Sequencing Data Processing and Sequence Analysis

Low-quality raw reads were eliminated using Trimmomatic (Bolger et al., 2014) based on the following criteria: (a) reads were truncated at any site receiving an average quality score < 20 over a 50 bp sliding window, and the truncated reads shorter than 50 bp; (b) 1 or more mismatch in barcode; (c) >2 nucleotide mismatch in primers. Paired-end reads were merged using FLASH (Magoč and Salzberg, 2011) with the parameter that overlap was longer than 10 bp and its mismatch rate was lower than 20%. Merged reads with length of >200 bp were kept and assigned to each sample based on the unique barcode (Caporaso et al., 2010; Bokulich et al., 2013). Chimera sequences were detected and removed using the UCHIME *de novo* algorithm (Edgar et al., 2011). Operational taxonomic units were clustered at a cut-off value of 0.97 similarity using USEARCH in the QIIME package (Caporaso et al., 2010; Edgar, 2010). A clustering value of 0.97 similarity was empirically confirmed by analyzing the clustering of taxonomical known *ureC* genes. Taxonomic assignment of representative sequences of OTUs was performed using GraftM^1^ with a likelihood cut-off of 0.75 when using pplacer (Matsen et al., 2010) for placement of the sequences against a compiled *ureC* gene package. The *ureC* gene package was compiled in graftM with the create command using a manually edited *ureC* alignment file. The alignment was generated from bacterial and archaeal *ureC* gene sequences with taxonomic assignment data which were downloaded from NCBI. The genes were aligned and manually edited using ARB software and then the region corresponding to the PCR amplicon was exported (Ludwig et al., 2004). Sequences containing more than 50% gaps in this region were removed with Belvue (Sonnhammer and Hollich, 2005). A phylogenetic tree was generated using FastTree (Price et al., 2009) in QIIME for calculating UniFrac distances. Alpha and beta diversity and significant fold changes of OTU’s were performed in the R packages ade4, Phyloseq, and DESeq2 (Chessel et al., 2004; McMurdie and Holmes, 2013; Love et al., 2014). The significances of grouping in the PCoA plots were tested by analysis of similarity (ANOSIM) with 999 permutations. Family level heatmap plots were generated in R using the ampvis R package (Albertsen et al., 2015), while annotated heatmaps of the top 50 OTUs were created using the NMF R package (Gaujoux and Seoighe, 2010).

### Statistical Analysis

The rumen NH_3_-N and urea-N concentration, urease activity, and diversity indices were analyzed using the SAS mixed procedure (SAS Institute, Inc, Cary, NC, USA) as shown in the following equation: Y_ijkl_ = μ + t_i_ + b_k_ + c(b)_jk_ + p_l_ + e_ijkl_, where Y_ijkl_ is the observation on cow j with treatment i, order of treatment k and period l; μ is the overall mean; t_i_ is the fixed effect of treatment i; b_k_ is the effect of order k of applying treatments; c(b)_jk_ is the random effect of cow j within order k; p_l_ is the effect of period l; and e_ijkl_ is the random error. Differences were declared significant at *P* < 0.05.

### Nucleotide Sequence Accession Number

All the raw sequences after assembling and filtering were submitted to the NCBI Sequence Read Archive (SRA^2^), under accession number SRP076839.

## Results

### Urea Metabolism in the Rumen

Urea supplementation significantly increased (*P* < 0.05) rumen NH_3_-N concentration at 2 and 4 h after morning feeding with the peak at 2 h (**Table 1**). No significant difference in the urease activity was observed between the control and urea groups, with both exhibiting maximum activity 2 h after feeding (*P* > 0.05). For the urea supplemented group, the increased urease activity at 2 h also coincided with higher NH_3_-N concentration.

**Table 1:** NH_3_-N and urea nitrogen (urea-N) concentrations and urease activity in the rumen of dairy cows from different treatments.

Item	Time (h)	Treatment		SEM	*P*		
		Control	Urea		Treatment	Period	Treatment × Period
NH_3_-N concentration (μmol dL^-1^)	0	18.16	21.95	1.550	0.24	0.07	0.67
	2	15.56^b^	31.05^a^	1.747	<0.01	0.58	0.22
	4	10.65^b^	23.81^a^	2.132	0.03	0.90	0.96
	6	8.61	14.32	1.148	0.1	0.78	0.49
Urea-N concentration (mg L^-1^)	0	4.90	6.60	1.092	0.28	0.51	0.28
	2	5.16	5.33	0.195	0.54	<0.01	0.14
	4	5.56	5.59	0.097	0.86	0.08	0.06
	6	5.52	5.58	0.156	0.76	0.05	0.45
Urease activity (nmol min^-1^ mg^-1^)	0	53.24	58.16	3.999	0.54	0.52	0.99
	2	61.37	62.32	10.397	0.97	0.75	0.80
	4	41.56	44.62	6.867	0.79	0.51	0.92
	6	33.59	31.62	6.204	0.73	0.40	0.50

*^a,b^ Different letters in the same row indicate statistically significant differences for treatment effect at *P* < 0.05.*

### Comparison of *ureC* Gene Diversity and Distribution

In total, 1,059,496 quality sequence reads were obtained with an average read length of 299 bases from the 36 samples. The total number of reads from each sample varied from 20,591 to 39,908 and the average reads number was 29,430. The total sequences were assigned to 588 OTUs using a cut-off of 97% sequence similarity.

**FIGURE 1:**
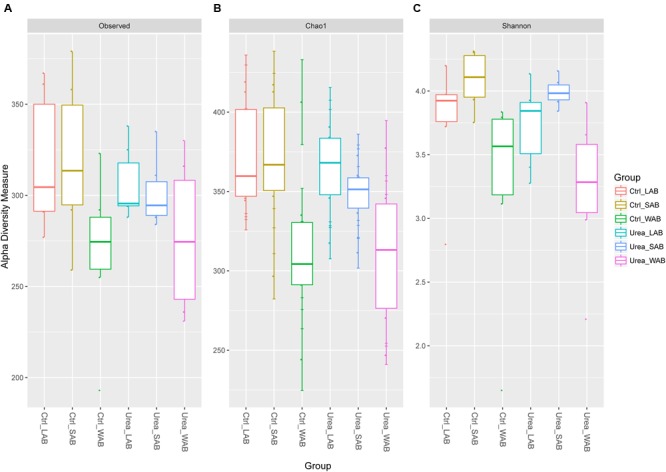
**Alpha diversity measures for *ure*C rumen microbiomes across different treatments and fractions. (A)** Total observed taxonomic units, **(B)** Chao1 estimates and, **(C)** the Shannon diversity index. Boxplots indicate the first and third quartiles with the median value indicated as a horizontal line the whickers extend to 1.5 times the inter quartile range. LAB, liquid-associated bacteria; SAB, solid-adherent bacteria; WAB, wall-adherent bacteria; Urea, urea group; Ctrl, control group.

Alpha diversity estimates are summarized in **Figure 1** and Supplementary Table S2. The total number of observed OTUs from the WAB was lower compared to the LAB and SAB fractions (*P* < 0.001). Good’s coverage estimates of sampling completeness showed greater than 99% coverage (Supplementary Table S2). Similar values for estimator Chao1, Shannon and Simpson indices were obtained for bacterial samples from the control and urea groups in each rumen fraction (*P* > 0.05), demonstrating no significant difference of the diversity measure and evenness of *ureC* genes after exogenous urea was provided to dairy cows. The Shannon diversity index for the WAB was lower than for the LAB and SAB fractions (*P* = 0.002).

**FIGURE 2:**
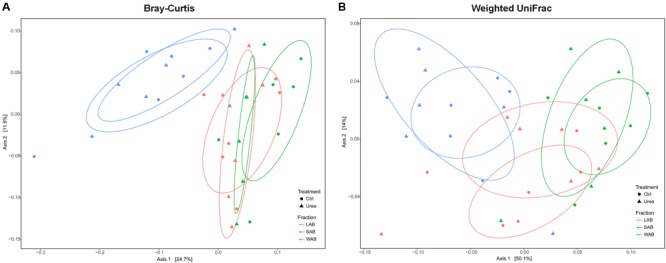
**Principle coordinate analysis comparing changes in rumen *ureC* genes based on Bray–Curtis (A)** and weighted Unifrac distances **(B)**. LAB, liquid-associated bacteria (red); SAB, solid-adherent bacteria (green); WAB, wall-adherent bacteria (blue); Urea, urea group (triangle); Ctrl, control group (circle).

The community composition of ureolytic microbiome as assessed by beta diversity measures demonstrated that the bacterial *ureC* gene composition of the WAB was significantly different from LAB and SAB fractions, with approximately 36 and 64% of the variance explained for the Bray–Curtis and weighted UniFrac metrics, respectively (Bray–Curtis, *R*^2^ = 0.198, *P* = 0.001; Weighted UniFrac, *R*^2^ = 0.343, *P* = 0.001) (**Figure 2**). However, there was no significant differences in bacterial community composition based on *ure*C genes between urea treated and control animals (Bray–Curtis, *R*^2^ = 0.015, *P* = 0.906; Weighted UniFrac, R^2^ = 0.010, *P* = 0.791).

**FIGURE 3:**
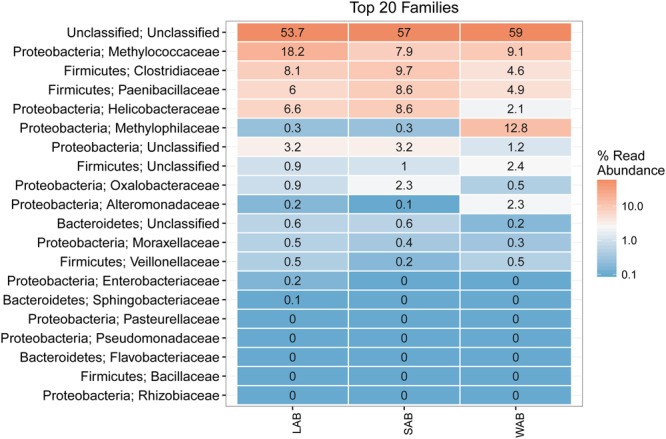
**Heatmap of the top 20 *ureC* gene families from different rumen fractions.** Taxonomic assignment shows the phylum and family level for each row. Numbers and color scale in cells represent the relative abundance for a given family level. LAB, liquid-associated bacteria; SAB, solid-adherent bacteria; WAB, wall-adherent bacteria.

Approximately 55% of the total sequences could not be confidently classified to any known phylum, while the remaining sequences were assigned to seven bacterial phyla. The majority of sequences were assigned to *Proteobacteria* (22.4–31.9%, SEM = 0.015), *Firmicutes* (11.1–20.2%, SEM = 0.014) and *Bacteroidetes* (0.2–0.8%, SEM = 0.001) from the different treatment groups and rumen fractions (**Figure 3**). At the family level, the dominant classified *ureC* genes in the rumen contents were from Methylococcaceae, Clostridiaceae, Paenibacillaceae, Helicobacteraceae, and Oxalobacteraceae while Methylophilaceae and Methylococcaceae were predominant in the WAB fraction (**Figure 3**). Interestingly, a very small number of *ureC* genes were affiliated with archaea from the Thaumarchaeota (0.0007%).

**FIGURE 4:**
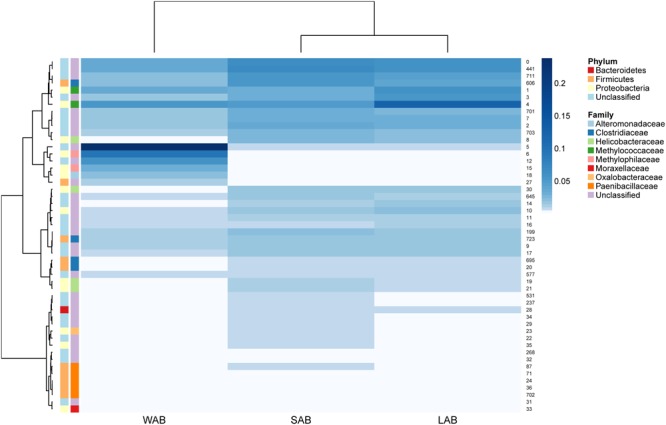
**Rumen *ureC* gene community heatmaps and clustering of the most abundant 50 operational taxonomic units (OTUs) from different rumen fractions.** Ward’s minimum variance method was used for hierarchical clustering of the computed distance matrix for samples based on the Jaccard dissimilarity indices of the OTU data in the vegan package. LAB, liquid-associated bacteria; SAB, solid-adherent bacteria; WAB, wall-adherent bacteria.

**FIGURE 5:**
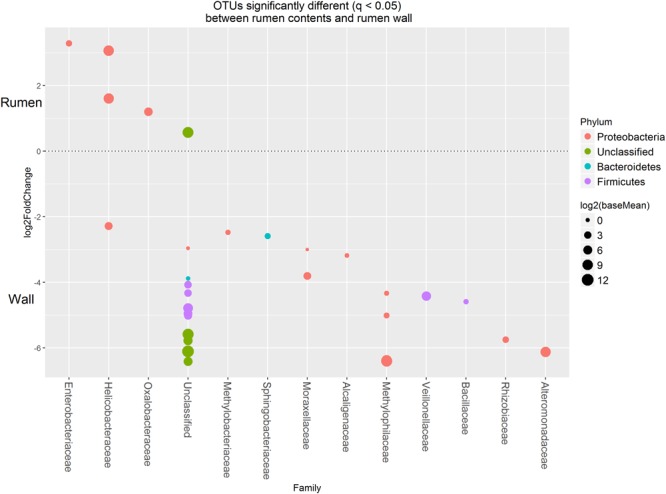
**Operational taxonomic units significantly different (*q* < 0.05 FDR) between the rumen contents (liquid and solid fractions) and the rumen wall.** Upper axis represents OTU’s with a log2 fold positive change for rumen contents relative to the rumen wall while the lower *y* axis is the negative fold change of the rumen wall relative to the rumen contents. Each point represents a single OTU colored by phylum and grouped on the *x* axis by taxonomic family level, size of point reflects the log2 mean abundance of the sequence data.

Approximately 85% of the sequence data was attributed to the top 50 abundant *ureC* gene OTUs. A high degree of similarity was observed for the rank abundance of OTUs for LAB and SAB, which clustered together and were distinct from the WAB fraction (**Figure 4**). A cluster of OTUs (5, 6, 12, 15, 18, and 27) exhibited higher rank abundance in the WAB and were absent or of lower abundance in the other two fractions. All of these OTUs were found to be significantly more abundant in the WAB (adjusted *p* < 0.001) (**Figure 5**). Two of the most abundant WAB OTUs, 5 and 12 were unclassified. Both OTU 6 and 15 were affiliated with the *Methylophilus* genus, and OTU 18 was classified with *Marinobacter*. A moderately abundant OTU 72 was classified to the Veillonellaceae family and a low abundant Helicobacteraceae OTU was also significantly linked with the WAB. The cluster which contained OTU 0, 441, 711, 606, 1, 3, and 4 was more abundant in the LAB and SAB compared to the WAB, but was seen consistently across all samples and was not significantly different. Both OTU 1 and 4 were affiliated with *Methylomonas* genus of bacteria. The *ureC* gene OTU 8, 30, 19, and 21 which affiliated with *Helicobacter* were most abundant in the rumen content (**Figure 4**), with OTUs 8 and 30 being significantly different from the WAB fraction (**Figure 5**).

## Discussion

Previous studies using culture-dependent methods have revealed limited information with regard to the rumen urea-degrading bacteria (Kakimoto et al., 1989; Lauková and Koniarová, 1994). By using the *ureC* gene as a biomarker for phylogenetic analysis we have obtained a better estimate of the composition of the ureolytic bacteria found in the rumen. Importantly, only about 45% of the sequences obtained could be assigned to any known phylum, indicating that the rumen may contain newly undiscovered sources of urease genes. Furthermore, the reference dataset used for taxonomic assignment was predominated by sequences from the Firmicutes and Proteobacteria phyla and will produce higher likelihood values for environmental sequences closely related to these species.

Urease genes from *Proteobacteria* constituted the highest proportion of classified sequences in all rumen samples which is in accord with studies from other environments, where urea-degrading microorganisms in open ocean and estuarine planktonic communities were mainly affiliated with this phylum (Collier et al., 2009). In our study, the *ureC* gene OTUs which belonged to rumen wall adherent bacteria were predominately from unclassified taxa, while some were affiliated with *Methylophilus* and *Marinobacter* bacteria. Methylotrophic species of bacteria from the genus Methylophilus (*M. methylotrophus, M. quaylei* sp. nov., and *M. rhizosphaerae* sp. nov.) with urease activity have been identified in studies from sludge and river water. These groups of bacteria can use methyl compounds such as methanol and methylamines for the assimilation of ammonia into cell protein (Greenwood et al., 1998; Doronina et al., 2005; Madhaiyan et al., 2009). An active-transport system for short-chain amides and urea has been identified in *M. methylotrophus* (Mills et al., 1998). *Marinobacter* species from marine environments are efficient degraders of aliphatic and polycyclic aromatic hydrocarbons as well as acyclic isoprenoid compounds (Duran, 2010). Genomic analysis of *Marinobacter aquaeolei* indicates this bacterium has the metabolic potential to utilize oxygen and nitrate as terminal electron acceptors, iron as an electron donor, and urea, phosphonate, and various hydrocarbons as alternative N, P, and C sources, respectively (Singer et al., 2011).

Urease genes with closest affiliation to *Helicobacter* spp. and *Methylomonas* spp. were present in all rumen sample fractions but were in higher abundance in the rumen contents. Previously, Zhao et al. (2015) had attempted to examine *ureC* diversity in the rumen digesta, by cloning and sequencing *ureC* genes, and found that among the total 317 *ureC* sequences, 22% were affiliated with *H. pylori* (98–100% aa sequence identity). The data from this study indicate that greater diversity and other taxonomic groups of ureolytic bacteria are more abundant in the rumen than *Helicobacter*. *Helicobacter* spp. naturally colonize the lining of stomach and intestines in human and animals (Fox, 2002; Harper et al., 2003), and they produce urease to maintain a neutral pH in their immediate environment. Some *Helicobacter* species isolated from the gastrointestinal tracts of sheep and dolphins have tested positive for urease activity (Harper et al., 2002; Coldham et al., 2011).

Among the predominant OTUs, both OTU 4 and 1, which were dominant in the rumen liquid fraction were affiliated with the Methylococcaceae family. Previous studies in aquatic environments have demonstrated that some *Methylomonas* spp. (*M. methanica, M. fodinarum*, and *M. paludis*) all possess urease activity (Dianou and Adachi, 1999; Boden et al., 2011). It is known that species of *Methylomonas* are able to obtain carbon and energy from oxidation of methane or methanol and use urea as a nitrogen source (Hoefman et al., 2014; Soren et al., 2015). Our results indicate that the ureolytic bacteria from the *Helicobacter* and *Methylomonas* that inhabit the rumen likely play an important role in hydrolyzing endogenous or exogenous urea.

Urea supplementation had no significant effect on the diversity and distribution of the *ureC* genes which was unexpected. The lack of response may be due to several factors. Firstly, the crude protein (CP) content (16.67% of DM) in the basal diet may have provided adequate ammonia, amino acid, or peptide for the synthesis of microbial protein (Agle et al., 2010; Recktenwald et al., 2014), and the bacteria may have used organic forms of nitrogen in preference to ammonia for the microbial protein synthesis (Milton et al., 1997; Lebzien, 2006). The regulation of urease synthesis in ureolytic bacteria is complex (Mobley et al., 1995), urease synthesis in some bacteria is regulated by environmental conditions, such as concentration of urea and nitrogen or pH (Collins and D’Orazio, 1993; Weeks and Sachs, 2001). However, in some organisms, urease synthesis is constitutive (Zotta et al., 2008; Carter et al., 2009; Burbank et al., 2012). Though the NH_3_-N concentrations in the urea supplemented group were higher than those in the control group, no differences in the urease activity between the two groups were observed. The conversion of urea to ammonia is rapid and not rate limiting, so on a high protein diet sufficient endogenous urea may have induced urease activity to an extent where differences did not occur between the two treatments even though urea and NH_3_-N concentrations might be higher in the urea supplemented group. Besides, Greenwood et al. (1998) also found that the urease was repressed by excess amounts of its reaction product, ammonia. Collectively all these factors may have contributed to the similar urease activity between the two treatments. Thus, the rumen harbors a large diversity of ureolytic bacteria but the mechanisms controlling urease synthesis and the impact of urea hydrolysis on the growth of these bacteria need further research.

## Conclusion

There was a predominant ureolytic bacterial community in the rumen of dairy cows but more than 55% of the *ureC* sequences did not affiliate with any known urease genes. The bacterial urease gene profile from the rumen wall was distinctly different from the rumen contents and *ureC* genes from Methylophilus and Marinobacter were identified predominantly in this fraction. The ureolytic bacterial populations were not changed in diversity or abundance by urea supplementation. This study contributes new data to existing urease gene information relating to the predominant ureolytic microbial community in ruminants. Understanding the rumen predominant urease genes may provide basis for acquiring valid regulation targets of ureolytic bacteria to mitigate urea hydrolysis and subsequently improve Urea-N utilization in ruminants.

## Author Contributions

JW, DB, and SZ designed the experiments; DJ performed the experiments; DJ, SZ, and SD analyzed the data; DJ wrote the paper; CM, SZ, NZ, and YB revised the paper. All authors agree to be accountable for all aspects of the work.

## Conflict of Interest Statement

The authors declare that the research was conducted in the absence of any commercial or financial relationships that could be construed as a potential conflict of interest.

## Footnotes

1 https://github.com/geronimp/graftM

2 http://www.ncbi.nlm.nih.gov/Traces/sra/
