# Supplementary material for: Differences in Ureolytic Bacterial Composition between the Rumen Digesta and Rumen Wall Based on ureC Gene Classification
Source: Front Microbiol. 2017 Mar 7;8:385. doi: 10.3389/fmicb.2017.00385 (PMC5339240; doi:10.3389/fmicb.2017.00385)
Supplement: Supplementary file 1 [file Data_Sheet_1.DOCX]

Supplementary Material

Differences in ureolytic bacterial composition between the rumen digesta and rumen wall based on *ureC* gene classification

Di Jin, Shengguo Zhao, Nan Zheng, Dengpan Bu, Yves Beckers, Stuart E. Denman, Christopher S. McSweeney and Jiaqi Wang^*^

*** Correspondence:** Jiaqi Wang: jiaqiwang@vip.163.com

# Supplementary Tables

Supplementary Table S1. Composition and nutrient levels of basal diets (air-dry basis)

Supplementary Table S2. Alpha diversity indices for the rumen bacteria *ureC* genes from each treatment group and rumen fraction.

**Supplementary Table S1. Composition and nutrient levels of basal diets (air-dry basis)**

| **Item** | **Content (%)** |
| --- | --- |
| Ingredients |  |
| Chinese wild rye | 3.7 |
| Alfalfa hay | 28.4 |
| Corn silage | 26.7 |
| Corn | 22.6 |
| Soybean meal | 11.8 |
| Cottonseed fuzzy | 5.1 |
| CaHPO_4_ | 0.6 |
| NaCl | 0.5 |
| Premix^a^ | 0.6 |
| Nutrient levels, % of DM |  |
| Crude protein (CP) | 16.7 |
| Ether Extract (EE) | 2.2 |
| Neutral detergent fiber (NDF) | 44.2 |
| Acid detergent fiber (ADF) | 26.1 |
| Ash | 7.7 |
| Ca | 0.8 |
| P | 0.3 |

^a^ One kilogram of premix DM contained the following: VA, 2,000,000 IU; VD, 600,000 IU; VE, 10,800 mg; Fe, 5,500 mg; Cu, 4,080 mg; Mn, 4,989 mg; Zn, 17,500 mg; I, 180 mg; Se, 110 mg; Co, 8,805 mg.

**Supplementary Table S2. Alpha diversity indices for the rumen bacteria *ureC* genes from each treatment groups and rumen fraction.**

| **Indices** | **Control** | | |  | **Urea** | | | **SEM** | ***P*** | | |
| --- | --- | --- | --- | --- | --- | --- | --- | --- | --- | --- | --- |
|  | **LAB** | **SAB** | **WAB** |  | **LAB** | **SAB** | **WAB** |  | **Trt** | **Fraction** | **Trt***  **Fraction** |
| Observed | 317^a^ | 319^a^ | 268^bc^ |  | 306^ab^ | 301^abc^ | 277^abc^ | 12.30 | 0.694 | <0.001 | 0.394 |
| Good’s coverage | 0.9963^c^ | 0.9968^abc^ | 0.9970b^a^ |  | 0.9965^bc^ | 0.9970^ab^ | 0.9971^a^ | <0.001 | 0.355 | 0.012 | 1.000 |
| PD | 20.91 | 19.52 | 19.02 |  | 20.75 | 19.18 | 19.85 | 0.298 | 0.857 | 0.087 | 0.682 |
| Chao1 | 373.52^a^ | 367.88^a^ | 314.19^bc^ |  | 362.37^ab^ | 347.23^ab^ | 311.09^c^ | 13.12 | 0.544 | <0.001 | 0.776 |
| Shannon | 3.756^ab^ | 4.086^a^ | 3.254b^c^ |  | 3.378^ab^ | 3.991^a^ | 3.222^bc^ | 0.125 | 0.790 | 0.002 | 0.978 |
| Simpson | 0.938^ab^ | 0.968^a^ | 0.875^ab^ |  | 0.942^ab^ | 0.965^ab^ | 0.869^b^ | 0.014 | 0.939 | 0.023 | 0.989 |

^a, b, c^ Different letters among various treatment groups and fractions indicate statistically significant differences (*P* < 0.05).

Observed, observed taxonomic units. PD, phylogenetic diversity; LAB, liquid-associated bacteria; SAB, solid-adherent bacteria; WAB, wall-adherent bacteria.
